# Nonlinear connection between remnant cholesterol and stroke risk: evidence from the China health and retirement longitudinal study

**DOI:** 10.1186/s12944-023-01943-8

**Published:** 2023-10-25

**Authors:** Yuanqing Wang, Fubing Zha, Yong Han, Ying Cai, Miaoling Chen, Cui Yang, Xiaodong Cai, Haofei Hu, Changchun Cao, Jiao Luo

**Affiliations:** 1 Department of Functional Neurology, The First Affiliated Hospital of Shenzhen University, Shenzhen Second People’s Hospital, Shenzhen, 518035 Guangdong China; 2 Department of Rehabilitation, The First Affiliated Hospital of Shenzhen University, Shenzhen Second People’s Hospital, Shenzhen, 518035 Guangdong China; 3 Department of Emergency, The First Affiliated Hospital of Shenzhen University, Shenzhen Second People’s Hospital, Shenzhen, 518035 Guangdong China; 4 Department of Rehabilitation, Shenzhen Dapeng New District Nan’ao People’s Hospital, No. 6, Renmin Road, Dapeng New District, Shenzhen, 518000 Guangdong Province China; 5 Department of Nephrology, The First Affiliated Hospital of Shenzhen University, Shenzhen Second People’s Hospital, No.3002, Sungang West Road, Futian District, Shenzhen, 518000 Guangdong Province China

**Keywords:** Stroke, Remnant cholesterol, Nonlinear relationship, Generalized additive model, Smoothed curve fitting

## Abstract

**Objective:**

The evidence on the relationship between remnant cholesterol (RC) and stroke remains controversial. Therefore, this study aimed to explore the relationship between RC and stroke risk in a Chinese population of middle-aged and elderly individuals.

**Methods:**

The present study included 10067 Chinese subjects of middle-aged and elderly individuals. The connection between RC and incident stroke was investigated using the multivariate Cox proportional hazards regression model, several sensitivity analyses, generalized additive models, and smoothed curve fitting.

**Results:**

A total of 1180 participants with stroke were recorded during the follow-up period. The multivariate Cox proportional hazards regression model identified a positive connection between RC and stroke risk (hazard ratio (HR) = 1.087, 95% confidence interval (CI): 1.001–1.180). In addition, the current study discovered a nonlinear connection between RC and incident stroke, and the point of inflection for RC was 1.78 mmol/L. The risk of stroke increased by 25.1% with each unit increase in RC level when RC was < 1.78 mmol/L (HR:1.251, 95%CI: 1.089–1.437, *P* = 0.0015). The results were not affected by sensitivity tests.

**Conclusion:**

The current study showed a positive and nonlinear connection between RC and stroke risk in a middle-aged and elderly Chinese population. These findings provided new information to help researchers better understand the relationship between RC levels and incident stroke.

**Supplementary Information:**

The online version contains supplementary material available at 10.1186/s12944-023-01943-8.

## Introduction

Stroke is the most common cause of disability and death and is a worrisome worldwide health problem [1]. Despite a continuous decline in age-standardized stroke mortality rates, the high stroke frequency continues to place a significant economic and health burden. There were 12.2 million incident strokes, 6.55 million deaths from stroke, and 143 million disability-adjusted life-years (DALYs) due to stroke, according to epidemiological data on the global disease burden in 2019 [2]. The risk of stroke rises progressively with age and may reach 69% in people over 65 years [3]. Therefore, identifying and managing risk factors for stroke is an effective and economical strategy for primary stroke prevention.

Remnant cholesterol (RC) is the amount of cholesterol found in intermediate-density lipoproteins, triglyceride-rich remnant lipoproteins, very low-density lipoproteins, and chylomicron remnants. Since RC carries most of the triglycerides in the plasma, RC concentration is strongly related to the triglyceride level, and a high triglyceride level is a sign of high RC [4]. In recent years, growing evidence has suggested that RC is the key factor in all-cause mortality and cardiovascular diseases (CVD) [5, 6]. However, the relationship between RC and stroke risk is still controversial. Some researchers have confirmed that RC is highly related to stroke risk [7, 8]. However, some evidence has indicated no significant correlation between RC and stroke [9–12]. In addition, almost no research has revealed the nonlinear relationship between RC and stroke, nor has subgroup analysis been used to evaluate the relationship between RC and stroke events.

The relationship between RC and stroke has sparked controversy in light of the studies above. Furthermore, when employing the Cox proportional hazards regression method to explore this connection, it becomes apparent that a nonlinear relationship could potentially influence the results. Therefore, we have formulated a hypothesis proposing the presence of nonlinearity between RC and stroke. In this context, the present study aimed to assess the influence of RC on the development of stroke among middle-aged and elderly Chinese adults by utilizing data from the China Health and Retirement Longitudinal Study (CHARLS).

## Methods

### Data source and study population

**Fig. 1:**
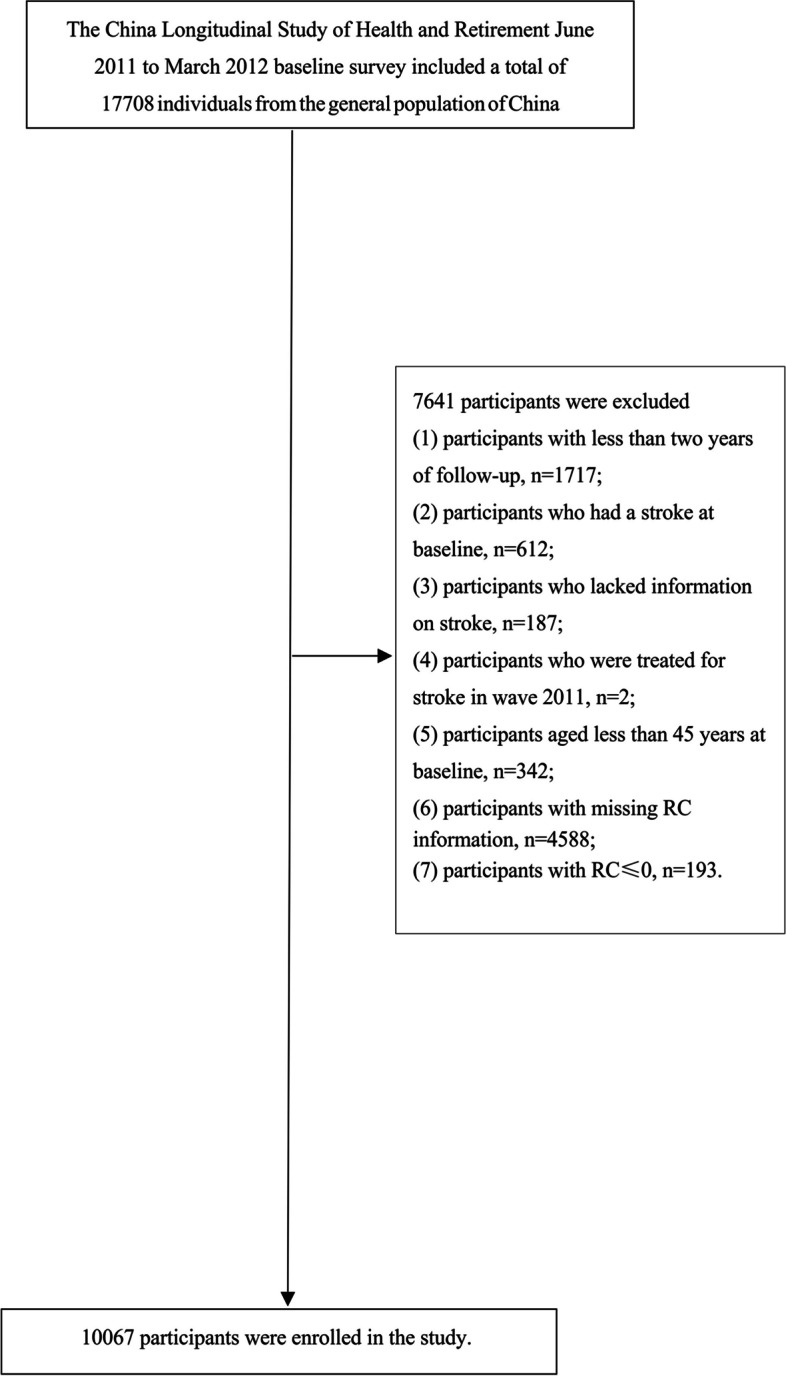
Study population

The CHARLS is a population-based, continuous longitudinal cohort study conducted nationally to assess social, health status, and economic factors [13]. The CHARLS provided the data for the current research. With 10257 homes and 17708 subjects participating in the baseline survey, the CHARLS sample was obtained from 450 communities in 150 districts, 28 provinces, and multi-stage probability sampling [13]. The sample was taken from the general retired population in China between June 2011 and March 2012 [13]. A longitudinal, face-to-face, computer-assisted personal interview was employed biennially to interact with the participants in the CHARLS [13]. Following the baseline survey from 2011 to 2012, survivors underwent three additional follow-up assessments from 2013 to 2014 (Wave 2), 2015 to 2016 (Wave 3), and 2017 to 2018 (Wave 4) [13]. The CHARLS website (http://charls.pku.edu.cn/en) has detailed information about the data [13]. Follow-up of fewer than two years, age < 45 years at baseline, lack of information on RC at baseline, history of stroke, lack of information on stroke at baseline, or treatment for stroke at baseline were exclusion criteria for the 17,708 patients enrolled in the study at the beginning. In the end, a total of 10067 subjects were included in the follow-up investigation (Fig. 1).

### Data collection

The CHARLS personnel at Peking University taught interviewers to use the computer-assisted personal interview method to conduct interviews in respondents' homes. Demographics, health conditions and functioning, chronic diseases diagnosed by a doctor, lifestyle, and health-related habits (drinking, physical activity, and smoking) were all included in the core CHARLS questionnaire [13]. In addition, to measure the health performance and functioning of the interviewees, the interviewers had the equipment to measure their blood pressure, height, and weight. Participants were invited to the township hospital or the nearby Centers for Disease Control and Prevention (CDC) office, where a skilled nurse took an 8 ml fasting blood sample. Complete blood count (CBC) testing was done between one and two hours after the sample was taken. While the CBC was done, the remaining blood was divided into plasma and blood cells and transported in a -20°C environment. For CDC analysis, all blood samples were shipped back to Beijing [13]. Diabetes mellitus was characterized by having fasting plasma glucose (FPG) levels ≥ 7.0 mmol/L, hemoglobin A1c (HbA1c) levels ≥ 6.5% [14], or a self-reported history of diabetes. Heart diseases were defined as myocardial infarction, coronary heart disease, congestive heart failure, or other heart diseases [13]. Hypertension was defined as blood pressure greater than 140/90 mmHg (average of 3 measurements) or a history of hypertension [13]. Physical activity was categorized as vigorous or moderate activity for more than 30 min at least once a week [13]. Participants who had previously smoked were defined as ever smokers, and those who still smoked were defined as current smokers [13]. Participants who reported that they had consumed alcoholic beverages in the past were defined as ever drinkers, and those who had consumed any alcoholic beverages within the last year were defined as current drinkers [13]. Participants with less than two years of follow-up (i.e., participants who did not receive any follow-up) were considered as lost to follow-up. Participants who were not followed up to Wave 4 (2017–2018) and did not reach the endpoint but were followed up for more than two years were defined as censorship.

### Covariates assessments

Based on a combination of prior research and clinical expertise, covariates were chosen [13, 15, 16]. The subsequent factors were used as covariates: (i) categorical variables: diabetes mellitus, sex, hypertension, smoking status, drinking status, physical activity, and lipid therapy; (ii) continuous variables: age, serum creatinine (Scr), C-reactive protein (CRP), and serum cystatin C.

### Assessment of remnant cholesterol level

The following is a description of the precise measurement process for RC: RC = total cholesterol (TC) (mmol/L)—low-density lipoprotein cholesterol (LDL-C)—high-density lipoprotein cholesterol (HDL-C) [17, 18].

### Diagnosis of stroke

The outcome variable was incident stroke throughout the follow-up period. As noted before, the following standard query was used to collect information on incident stroke: "Have you been informed by a doctor that you have a stroke diagnosis?" or "Because of your stroke, are you presently receiving any of the following treatments: taking Western medicine, physical therapy, taking Chinese traditional medicine, acupuncture therapy, and occupational therapy" [13]. If the individual provided a positive response at follow-up, the respondent was classified as having a first stroke diagnosis, and the self-reported time was recorded as the onset of the stroke. The time of the event was determined by subtracting the time of the baseline survey from the time of stroke onset.

### Missing data processing

It is difficult to completely avoid missing data in observational research, which is a common issue [19]. There were 8 (0.08%), 1502 (14.92%), 91 (0.90%), 54 (0.54%), 11 (0.11%), 5996 (59.56%), 169 (1.68%), 12 (0.12%), 13 (0.13%), 80 (0.79%), and 2464 (24.48%) individuals with missing data for gender, BMI, diabetes mellitus, hypertension, drinking status, physical activity, smoking status, FPG, Scr, HbA1c, and Cystatin C, respectively. Missing clinical variables were addressed via chained equations using multiple imputations for modeling purposes. Age, sex, BMI, diabetes mellitus, hypertension, drinking status, physical activity, smoking status, lipid therapy, FPG, Scr, HbA1c, CRP, triglyceride (TG), and Cystatin C were all incorporated in the imputation model, which employed linear regression and ten iterations. The assumption of missing at random was used in missing data analysis processes [19].

### Statistical analysis

All participants were divided into four groups based on RC quartiles (Q1 ≤ 0.310 mmol/L; 0.310 mmol/L < Q2 ≤ 0.520 mmol/L; 0.520 mmol/L < Q3 ≤ 0.84 mmol/L; Q4 > 0.84 mmol/L). The continuous baseline data were expressed as the mean ± the standard deviation (SD) (normally distributed data) and medians (quartile) (nonnormally distributed data). The current study used the expression in numbers (percentages) for categorical data. Comparisons were made using either ANOVA (nonnormally distributed data), the χ2 test (categorical data), or the Kruskal–Wallis test (skewed data). Incidence rates were expressed in person-years and cumulative incidence.

Using Cox proportional hazards models, the current study calculated the hazard ratios (HR) and 95% confidence intervals (CI) for stroke events. In addition, three Cox proportional hazards models were performed. The current study referenced prior research and clinical expertise in selecting covariates. The inclusion of these covariates in the model led to a substantial change of 10% or more in the HR. In Model 1, no covariates were adjusted. In Model 2, BMI, gender, age, drinking status, physical activity, heart diseases, diabetes mellitus, hypertension, and smoking status were controlled. BMI, gender, age, drinking status, physical activity, heart diseases, diabetes mellitus, hypertension, smoking status, lipid therapy, CRP, Scr, and cystatin C were used as adjustment variables of Model 3. Controlling variables were adjusted based on clinical knowledge and published reports [13, 15, 16]. Based on the results of the collinearity screening, no covariates were excluded from the Cox proportional hazards regression models according to the screening results for collinearity (Table S1).

The current study performed numerous sensitivity analyses to evaluate the reliability of the results. The current study converted RC into a categorical variable depending on the quartiles of the RC. By determining *P* for the trend, the results of RC as a continuous variable were examined, and the possibility of nonlinearity between RC and incident stroke was investigated. Hypertension and obesity are strong risk factors for stroke [20, 21]. Given their potential to confound the association between RC and incident stroke, we adopted an exclusionary approach in our analysis. Specifically, individuals with BMI ≥ 24 kg/m^2^ and hypertension were systematically excluded from our study cohort. This methodological decision was undertaken with the primary aim of elucidating and corroborating the relationship between RC and stroke while ensuring the stability and robustness of our findings. Furthermore, to minimize potential sources of bias and enhance the internal validity of our investigation, we extended the exclusion criteria to encompass non-hypertensive individuals with BMI < 24 kg/m^2^. These exclusions were conducted to foster a more homogenous study population and minimize the influence of confounding variables, thereby allowing for a more precise examination of the relationship between RC and stroke.

Moreover, the current study tested the nonlinear relationship between RC and stroke risk using the Cox proportional hazards regression model with cubic spline functions and the smooth curve fitting. If nonlinearity was observed, the current study's initial step involved utilizing a recursive algorithm to calculate the inflection point. The algorithm was initiated arbitrarily, and filtering and smoothing techniques were employed to identify the inflection point. Subsequently, a two-piece Cox proportional hazards regression model was constructed on either side of the inflection point. The log-likelihood ratio was employed to determine the most appropriate model for elucidating the association between RC and the risk of stroke.

All the results were reported under the STROBE statement [22]. All analyses were conducted using R statistical software tools and Empower Stats. The threshold for statistical significance (two-sided) was established at *P* values less than 0.05.

## Results

### Participants’ characteristics

In the present study, there were 10,067 participants (4697 men and 5370 women) with an age range of 45 to 99 years and a mean age of 59.148 ± 9.348 years. Following a median follow-up of 7.0 years, 1180 (11.72%) individuals developed stroke.

Table 1 listed the characteristics of the study population. RC was classified into four groups based on the quartiles (Q1 ≤ 0.310 mmol/L; 0.310 mmol/L < Q2 ≤ 0.520 mmol/L; 0.520 mmol/L < Q3 ≤ 0.84 mmol/L; Q4 > 0.84 mmol/L). The current study showed that participants generally had higher levels of BMI, FPG, Scr, HbA1c, TG, and higher proportions of diabetes mellitus, hypertension, and lipid therapy in the Q4 group. In addition, participants in the Q1 group had lower levels of Cystatin C and higher rates in males.

**Table 1:** The baseline characteristics of participants

Variables	**RC group (mmol/L)**				***P*-value**
	**Q1 (≤ 0.310)**	**Q2 (0.310 to ≤ 0.520)**	**Q3 (0.520 to ≤ 0.840)**	**Q4 (> 0.840)**	
Participants	2513	2502	2529	2523	
Gender					< 0.001
Male	1253 (49.861%)	1203 (48.082%)	1112 (43.970%)	1129 (44.748%)	
Female	1260 (50.139%)	1299 (51.918%)	1417 (56.030%)	1394 (55.252%)	
Age (years)	59.388 ± 9.686	59.555 ± 9.342	59.072 ± 9.192	58.580 ± 9.142	0.001
BMI (kg/m^2^)	22.526 ± 3.562	23.110 ± 3.818	23.715 ± 3.962	24.997 ± 4.103	< 0.001
Drinking status					0.083
Never drinkers	329 (13.092%)	347 (13.869%)	351 (13.879%)	354 (14.031%)	
Ever drinkers	1495 (59.491%)	1525 (60.951%)	1558 (61.605%)	1576 (62.465%)	
Current drinkers	689 (27.417%)	630 (25.180%)	620 (24.516%)	593 (23.504%)	
Smoking status					0.178
Never smokers	1545 (61.480%)	1517 (60.631%)	1583 (62.594%)	1584 (62.782%)	
Ever smokers	221 (8.794%)	207 (8.273%)	200 (7.908%)	235 (9.314%)	
Current smokers	747 (29.725%)	778 (31.095%)	746 (29.498%)	704 (27.903%)	
Physical activity					< 0.001
No	846 (33.665%)	866 (34.612%)	921 (36.418%)	1019 (40.388%)	
Yes	1667 (66.335%)	1636 (65.388%)	1608 (63.582%)	1504 (59.612%)	
Heart diseases					0.020
No	2222 (88.420%)	2236 (89.369%)	2216 (87.624%)	2185 (86.603%)	
Yes	291 (11.580%)	266 (10.631%)	313 (12.376%)	338 (13.397%)	
Lipid therapy					< 0.001
No	2439 (97.055%)	2403 (96.043%)	2409 (95.255%)	2314 (91.716%)	
Yes	74 (2.945%)	99 (3.957%)	120 (4.745%)	209 (8.284%)	
Hypertension					< 0.001
No	2027 (80.661%)	1946 (77.778%)	1909 (75.484%)	1724 (68.331%)	
Yes	486 (19.339%)	556 (22.222%)	620 (24.516%)	799 (31.669%)	
Diabetes mellitus					< 0.001
No	2411 (95.941%)	2379 (95.084%)	2405 (95.097%)	2316 (91.795%)	
Yes	102 (4.059%)	123 (4.916%)	124 (4.903%)	207 (8.205%)	
HDL-C (mmol/L)	1.551 ± 0.374	1.384 ± 0.349	1.281 ± 0.344	1.045 ± 0.304	< 0.001
TG (mmol/L)	0.800 ± 0.247	1.070 ± 0.309	1.418 ± 0.411	2.754 ± 1.519	< 0.001
LDL-C (mmol/L)	3.021 ± 0.825	3.079 ± 0.852	3.084 ± 0.871	2.838 ± 1.034	< 0.001
TC (mmol/L)	4.762 ± 0.916	4.873 ± 0.912	5.028 ± 0.944	5.347 ± 1.080	< 0.001
CRP (mg/L)	0.813 (0.469–1.720)	0.980 (0.540–2.170)	1.080 (0.563–2.250)	1.252 (0.660–2.550)	< 0.001
Scr (mg/dL)	0.773 ± 0.330	0.778 ± 0.210	0.777 ± 0.185	0.800 ± 0.200	< 0.001
FPG (mg/dL)	103.085 ± 24.337	105.621 ± 28.182	110.605 ± 41.044	121.122 ± 43.124	< 0.001
HbA1c (%)	5.151 ± 0.666	5.166 ± 0.685	5.274 ± 0.878	5.395 ± 0.945	< 0.001
Cystatin C (mg/L)	1.033 ± 0.329	1.028 ± 0.257	1.003 ± 0.244	0.949 ± 0.269	< 0.001

Values are n (%) or mean ± SD or median (quartile)

*RC* remnant cholesterol, *BMI* body mass index, *HDL-C* high-density lipoprotein cholesterol, *LDL-C* low-density lipoprotein cholesterol, *TC* total cholesterol, *TG* triglycerides, *Scr* serum creatinine, *CRP* C-reactive protein, *FPG* fasting plasma glucose, *HbA1c* glycosylated hemoglobin

### The incidence rate of stroke

The cumulative incidence rate of stroke is shown in Table 2. Stroke incidence rates were 11.72% (11.09%-12.34%), 9.63% (8.48%-10.78%), 11.15% (9.92%-12.39%), 11.70% (10.45%-12.96%), and 14.39% (13.02%-15.76%) respectively for the general population, Q1 group, Q2 group, Q3 group, and Q4 group. The respective incidence rates were 19.21, 15.67, 18.22, 19.14, and 23.89 per 1,000 person-years for all individuals and the four RC groups.

**Table 2:** Incidence rate of incident stroke

RC	Participants (n)	stroke events (n)	Incidence rate (95%CI) (%)	Per 1,000 person-years
Total	10067	1180	11.72 (11.09–12.34)	19.21
Q1	2513	242	9.63 (8.48–10.78)	15.67
Q2	2502	279	11.15 (9.92–12.39)	18.22
Q3	2529	296	11.70 (10.45–12.96)	19.14
Q4	2523	363	14.39 (13.02–15.76)	23.89

*RC* remnant cholesterol, *CI* confidence interval

### The risk factors for incident stroke

RC, age, BMI, female, current smokers, heart diseases, diabetes mellitus, hypertension, and lipid therapy were independent risk factors for stroke (Table 3). In addition, each unit increase in RC was linked to a 19.2% increase in the risk of stroke in Model 1 without adjusting for any variables (HR = 1.192, 95%CI: 1.108–1.282). The risk of stroke increased by 9.7% for every mmol/L rise in RC after adjusting for BMI, gender, age, drinking status, physical activity, heart diseases, diabetes mellitus, and hypertension in Model 2 (HR = 1.097, 95%CI: 1.014–1.188). Each unit of RC was associated with an 8.7% increase in stroke risk after adjusting for BMI, gender, age, drinking status, physical activity, heart diseases, diabetes mellitus, hypertension, lipid therapy, smoking status, CRP, Scr, and Cystatin C in Model 3 (HR = 1.087, 95%CI: 1.001–1.180). Additionally, the results revealed a significant association between the highest quartile (Q4) of RC and an increased risk of stroke when using the lowest quartile (Q1) as a reference (Model 1, Q4: HR = 1.520, 95%CI: 1.292–1.788) (Model 2, Q4: HR = 1.277, 95%CI: 1.080–1.510) (Model 3, HR = 1.261, 95%CI: 1.063–1.495) (Table 3).

**Table 3:** Relationship between RC and the incident stroke in different models

Exposure	Model 1 (HR.,95% CI, *P*)	Model 2 (HR,95% CI, *P*)	Model 3 (HR,95% CI, *P*)
RC	1.192 (1.108, 1.282) < 0.00001	1.097 (1.014, 1.188) 0.02200	1.087 (1.001, 1.180) 0.04675
RC (quartile)			
Q1	ref	ref	ref
Q2	1.159 (0.976, 1.377) 0.09240	1.142 (0.961, 1.357) 0.13243	1.137 (0.957, 1.352) 0.14386
Q3	1.219 (1.028, 1.444) 0.02244	1.134 (0.956, 1.346) 0.14907	1.130 (0.952, 1.341) 0.16290
Q4	1.520 (1.292, 1.788) < 0.00001	1.277 (1.080, 1.510) 0.00431	1.261 (1.063, 1.495) 0.00777
Gender			
Male		ref	ref
Female		1.162 (0.989, 1.365) 0.06746	1.193 (1.005, 1.415) 0.04370
Age (years)		1.021 (1.015, 1.028) < 0.00001	1.020 (1.013, 1.027) < 0.00001
BMI (kg/m^2^)		1.022 (1.008, 1.036) 0.00240	1.019 (1.005, 1.034) 0.00935
Drinking status			
Never drinkers		ref	ref
Ever drinkers		0.891 (0.751, 1.057) 0.18545	0.893 (0.753, 1.060) 0.19543
Current drinkers		0.870 (0.719, 1.052) 0.15021	0.876 (0.724, 1.060) 0.17200
Smoking status			
Never smokers		ref	ref
Ever smokers		1.224 (0.991, 1.512) 0.06115	1.217 (0.984, 1.505) 0.06965
Current smokers		1.240 (1.050, 1.464) 0.01106	1.236 (1.047, 1.460) 0.01246
Physical activity			
No		ref	ref
Yes		0.901 (0.800, 1.014) 0.08424	0.906 (0.805, 1.021) 0.10586
Heart diseases			
No		ref	ref
Yes		4.059 (3.580, 4.603) < 0.00001	3.967 (3.494, 4.504) < 0.00001
Hypertension			
No		ref	ref
Yes		1.641 (1.445, 1.864) < 0.00001	1.605 (1.412, 1.826) < 0.00001
Diabetes mellitus			
No		ref	ref
Yes		1.418 (1.176, 1.710) 0.00025	1.363 (1.127, 1.647) 0.00137
Lipid therapy			
No			ref
Yes			1.281 (1.059, 1.550) 0.01077
CRP (mg/L)			1.006 (1.000, 1.013) 0.05031
Scr (mg/dL)			1.109 (0.801, 1.535) 0.53333
Cystatin C (mg/L)			1.035 (0.786, 1.363) 0.80615

Model 1 did not adjust for other covariants

Model 2 adjusted for BMI, gender, age, drinking status, physical activity, heart diseases, diabetes mellitus, hypertension, and smoking status

Model 3 adjusted for BMI, gender, age, drinking status, physical activity, heart diseases, diabetes mellitus, hypertension, smoking status, lipid therapy, CRP, Scr, and Cystatin C

*HR* hazard ratios, *CI* confidence interval, *Ref* reference, *RC* remnant cholesterol

### Sensitivity analyses

The sensitivity analysis excluding individuals with BMI ≥ 24 kg/m^2^ (Table 4, Model 4) revealed a positive connection between RC and incident stroke after adjusting for confounding covariates (HR = 1.178, 95%CI: 1.028–1.349). RC remained positively associated with incident stroke after performing sensitivity analyses including non-hypertensive (Table 4, Model 5) and non-hypertensive subjects with BMI < 24 kg/m^2^ (Table 4, Model 6). After adjusting for confounding covariates, the HR was 1.134, 95%CI: 1.018–1.263 (Table 4, Model 5) and 1.281, 95%CI: 1.091–1.504 (Table 4, Model 6).

**Table 4:** Relationship between RC and stroke risk in different sensitivity analyses

Exposure	Model 4 (HR, 95%CI, *P*)	Model 5 (HR, 95%CI, *P*)	Model 6 (HR, 95%CI, *P*)
RC	1.178 (1.028, 1.349) 0.01845	1.134 (1.018, 1.263) 0.02251	1.281 (1.091, 1.504) 0.00256
RC (quartile)			
Q1	ref	ref	ref
Q2	1.100 (0.873, 1.385) 0.41827	1.152 (0.920, 1.443) 0.21824	1.232 (0.932, 1.629) 0.14260
Q3	1.143 (0.908, 1.438) 0.25533	1.157 (0.922, 1.451) 0.20739	1.236 (0.928, 1.645) 0.14723
Q4	1.485 (1.170, 1.885) 0.00116	1.464 (1.167, 1.838) 0.00101	1.787 (1.330, 2.401) 0.00012

Model 4 was sensitivity analysis in participants without BMI ≥ 24 kg/m^2^. Model 4 adjusted for gender, age, drinking status, physical activity, heart diseases, diabetes mellitus, hypertension, smoking status, lipid therapy, CRP, Scr, and Cystatin C

Model 5 was sensitivity analysis in participants without hypertension. Model 5 adjusted for BMI, gender, age, drinking status, physical activity, heart diseases, diabetes mellitus, smoking status, lipid therapy, CRP, Scr, and Cystatin C

Model 6 was sensitivity analysis in participants without hypertension and BMI ≥ 24 kg/m^2^. Model 6 adjusted for gender, age, drinking status, physical activity, heart diseases, diabetes mellitus, smoking status, lipid therapy, CRP, Scr, and Cystatin C

*HR* hazard ratios, *CI* confidence, *Ref* reference, RC: remnant cholesterol

### The analysis of the nonlinear relationship

**Fig. 2:**
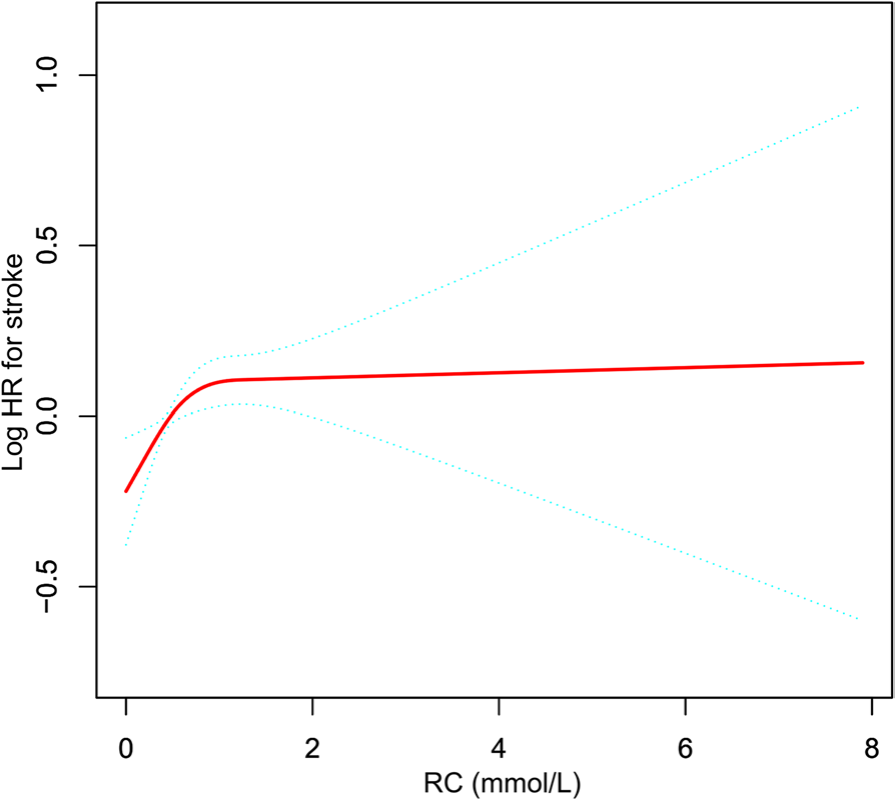
The nonlinear relationship between RC and incident stroke in Chinese individuals aged 45 years or older

Figure 2 presents the nonlinear relationship between RC and stroke risk. There was a non-linear correlation between RC and incident stroke after adjusting for confounding covariates (Table 5). The inflection point of RC was 1.78 mmol/L according to a two-piecewise Cox proportional hazards regression model (*P* for log-likelihood ratio test = 0.013). RC positively connected with the incident stroke when was RC < 1.78 mmol/L (HR:1.251, 95%CI: 1.089–1.437). In contrast, the connection between RC and incident stroke was not significant when RC ≥ 1.78 mmol/L (HR: 0.893, 95%CI: 0.736–1.084).

**Table 5:** The result of the two-piecewise Cox proportional hazards regression model

Incident stroke	HR (95%CI)	*P*
Fitting model by standard linear regression	1.087 (1.001, 1.180)	0.0467
Fitting model by two-piecewise Cox proportional hazards regression		
Inflection point of RC	1.78 mmol/L	
≤ 1.78 mmol/L	1.251 (1.089, 1.437)	0.0015
> 1.78 mmol/L	0.893 (0.736, 1.084)	0.2541
P for the log-likelihood ratio test	0.013	

This model adjusted for BMI, gender, age, drinking status, physical activity, heart diseases, diabetes mellitus, hypertension, smoking status, lipid therapy, CRP, Scr, and Cystatin C

*HR* hazard ratios, *CI* confidence, *RC* remnant cholesterol

## Discussion

In this nationwide prospective cohort study of Chinese individuals aged 45 or older, we discovered a positive and nonlinear connection between RC and stroke risk. The connection between RC and stroke risk was a saturated effect curve with a point of inflection at 1.78 mmol/L. RC was associated with stroke occurrence after adjustment for confounders when RC < 1.78 mmol/L. In addition, we found that age, BMI, female, current smokers, heart diseases, diabetes mellitus, hypertension, and lipid therapy were independent risk factors for stroke. Clinically, risk mitigation strategies for stroke encompass the management of multiple factors, including control of risk factors such as elevated RC. Furthermore, a multifaceted approach involving the management of age, BMI, gender (specifically, female), tobacco use (current smokers), pre-existing heart diseases, diabetes mellitus, hypertension, and lipid therapy can synergistically contribute to a reduction in the overall risk of stroke. When applied in concert, these risk-modifying interventions may yield a more comprehensive and effective stroke prevention strategy within the clinical setting.

In the overall Chinese population, the incidence of stroke recently reached 2.47 per 1,000 person-years [23]. However, the incidence of stroke in the current study was 19.21 per 1,000 person-years, which is significantly higher than reported in previous research [23]. This disparity could be attributed to the predominant inclusion of individuals aged 45 years or older in the current research population. Therefore, it was reasonable to assume that the current study participants had a much higher incidence of stroke than the general population. Therefore, it was imperative to explore potential risk factors for stroke in adults aged 45 years and older.

The results of many studies in the past showed that RC is a high-risk factor for CVD. However, there is still debate and uncertainty surrounding the connection between RC and incident stroke. In a Korean longitudinal cohort study that included 1956452 patients with type 2 diabetes without atherosclerotic CVD, the highest RC quartile was independent of the risk of stroke compared with the lowest quartile risk factor (HR: 1. 281, 95%CI: 1.249–1.314) after adjustment for age, smoking status, sex, duration of diabetes, BMI, fibrate use, statin use, regular exercise, low income, alcohol drinking status, hypertension, and FPG [7]. In another cohort study involving 106937 subjects, elevated RC levels were related to a higher risk of ischemic stroke (HR: 1.8, 95%CI: 1.4–2.5) after adjustment for age, sex, low-density lipoprotein cholesterol, smoking status, cumulative smoking, systolic blood pressure, birth year, and atherosclerotic cardiovascular disease before baseline [8]. However, other studies reported no association between RC and stroke risk. In a United States cohort study of 9334 participants without CVD, the results of the study showed no association between RC in the high-level group and the incidence of new strokes compared to RC in the low-level group after adjusting for potential confounders (HR = 1.07; 95%CI:0.78–1.45) [11]. A prospective cohort study including 8782 subjects aged 45 or older from rural northeastern China demonstrated that RC was not related to stroke risk (HR:1.25, 95%CI: 0.94–1.66) after adjusting for potential confounders [10]. Elevated RC was not linked to an increased risk of stroke in patients with acute coronary syndromes or myocardial infarction in nonobstructive coronary arteries [9, 12]. The current study strengthened the argument that a high RC is positively associated with stroke risk in the existing literature. The possible reasons for these inconsistent findings are differences in the gender ratio, study populations, RC range, and adjustment factors of these studies. In addition, it was noteworthy that the results of linear regression analysis might be influenced by nonlinear connections, resulting in variations in the fitted linear relationship. In other words, the divergent findings from these studies could be attributed to a nonlinear association between RC and incident stroke.

After categorizing the RC into quartiles and using the first quartile (Q1) as the reference in the current study (Table 3, Model 3), the hazard ratios for the second (Q2), third (Q3), and fourth (Q4) quartiles were 1.146, 1.137 and 1.288, respectively. The findings from the analysis of RC as a categorical variable demonstrated a gradual increase in the risk of stroke from Q1 to Q4. This result is consistent with the results using RC as a continuous variable, indicating a consistent and stable relationship between RC and the risk of stroke.

Besides, we discovered the nonlinear connection between RC and incident stroke, performing a two-piecewise Cox proportional hazards regression model with a saturation effect. The inflection point of RC was 1.78 mmol/L after adjustment for confounders. The results indicated that for every unit increase in RC level when it was < 1.78 mmol/L, the probability of stroke elevated by 25.1% (HR:1.251, 95%CI: 1.089–1.437). However, each unit increase in RC level was not associated with incident stroke when RC was ≥ 1.78 mmol/L (HR: 0.893, 95%CI: 0.736–1.084). Based on the results above, it could be inferred that a decrease in RC was associated with a reduced risk of stroke when RC was below the inflection point. Conversely, when RC was above the inflection point, changes in RC did not significantly affect the risk of stroke. In other words, reducing RC was likely to lower the risk of stroke only when RC was below the inflection point. Therefore, from a clinical perspective, lowering RC below the inflection point might be an effective strategy for reducing the risk of stroke. Differences in the relationship between RC and stroke incidence on either side of the inflection point might arise from other factors affecting stroke risk. Table S2 displayed that compared with RC < 1.78 mmol/L, subjects with RC ≥ 1.78 mmol/L typically had greater FPG, BMI, TG, Scr, and HbA1c. However, all of these indicators were risk factors for stroke [24], which might weaken the effect of RC on stroke risk. On the other hand, when RC was lower than 1.78 mmol/L, these risk factors (FPG, BMI, TG, Scr, and HbA1c) for stroke were reduced, and their influence on stroke was lessened, and RC had a considerably enhanced effect on stroke.

By inducing atherosclerosis, RC might theoretically account for the mechanical link between increased RC and an elevated risk of stroke [17]. Previous studies demonstrated that RC, such as very-low-density lipoproteins (VLDL), triglyceride-rich lipoproteins (TGLs), and intermediate-density lipoproteins (IDL), could get into and lock the arterial intima [25–28], while large chylomicrons and the largest VLDL particles were unable to get into the arterial intima because of the size of these particles [29–31]. Because of this, small-size lipoprotein particles had a higher potential to cause an atherogenic reaction in the artery intima. These particles became stuck and found it difficult to return to the artery lumen by interacting with proteoglycans and other elements of the arterial intima, leading to cholesterol buildup [28]. For them to be picked up by scavenger receptors on the surface of macrophages after being trapped, they would first need to be changed. This would result in the creation of foam cells and the growth of atherosclerotic plaque [32]. Lipolysis products produced by RC particles, including oxidized free fatty acids, trigger the synthesis of cytokines, interleukins, and atherogenic adhesion molecules and might cause local inflammation in the arterial wall [33]. Therefore, RC's primary atherogenic action was mostly brought on by the size of its particles, which resulted in a lengthened residence period in the artery intima, enhanced penetration into the arterial wall, and increased sensitivity to oxidation.

### Strengths

The current study presents the following strengths. First, this is the first study examining the connection between RC and incident stroke in subjects aged 45 or older. Second, the CHARLS project, a nationally representative cohort research with a high response rate, provided the data for this study. Third, the current study examined the nonlinear connection between RC and incident stroke. Previous studies explored the relationship between RC and stroke based on linear regression. And their results were inconsistent. Besides, a nonlinear relationship is a relationship between two variables in which change in one entity does not correspond with constant change in the other variable. This might mean the relationship between the two variables seems unpredictable or virtually absent. However, nonlinear entities can be related to each other in fairly predictable ways but are simply more complex than in a linear relationship because of the complexity of the relationship between RC and stroke. Therefore, the nonlinear relationship may also be closer to the relationship between RC and stroke risk. Fourth, residual confounding factors were minimized by using strict statistical adjustments. Fifth, the present study conducted sensitivity analyses to ensure the stability of the findings.

### Limitations

The present investigation has some restrictions. First, Chinese adults over 45 were selected for the current study. Therefore, additional testing of these findings for younger populations and other ethnicities is required. Second, the current study was unable to differentiate between the various stroke types. Therefore, it is still unclear whether there is a connection between RC, hemorrhagic stroke, and ischemic stroke. Third, certain unmeasured confounders, such as the family history of stroke and diet, may have impacted the connection between RC and stroke risks. Fourth, the small proportion of the population (only 5%) with an RC greater than 1.78 mmol/L in this study might impact the precision and generalizability of the results. Fifth, stroke diagnosis in this study was solely based on self-report, without considering historical information and imaging findings. Despite the lack of CHARLS validation data specifically for self-reported stroke diagnosis, previous stroke epidemiological studies have reported sensitivity and specificity rates ranging from 78 to 96% and 96% to 98% [34–36] when using self-report. Future research aims to develop comprehensive studies incorporating historical information and imaging findings to enhance the accuracy of stroke diagnosis.

## Conclusion

This current study revealed a positive and nonlinear connection between RC and stroke risk in a middle-aged and elderly Chinese population. A statistically significant positive correlation existed between stroke and RC levels lower than 1.78 mmol/L. Clinicians can use this finding as a guide to manage the RC level. Reducing the RC level below the inflection point makes sense from a therapy standpoint. When the RC level is below the inflection point, lowering it can considerably lower the risk of stroke.

### Supplementary Information

**Additional file 1:** **Table S1.** The results of the collinearity screening. **Table S2.** The Baseline Characteristics of participants based on inflection point for RC.

## Data Availability

Data are available from http://www.isss.pku.edu.cn/cfps/. Follow the prompts to register as a user and download the data once it has been reviewed and approved.

## Abbreviations

RC: Remnant cholesterol
DALYs: Disability-adjusted life-years
CVD: Cardiovascular diseases
CHARLS: China Health and Retirement Longitudinal Study
BMI: Body mass index
FPG: Fasting plasma glucose
TC: Total cholesterol
TG: Triglyceride
HDL-C: High-density lipoprotein cholesterol
LDL-C: Low-density lipid cholesterol
Non-HDL-C: Non-high-density lipoprotein cholesterol
Scr: Serum creatinine
HbA1c: Glycosylated hemoglobin
CRP: C-reactive protein
HR: Hazard ratios
CI: Confidence intervals
Ref: Reference
sdLDL-C: Small dense low-density lipoprotein-cholesterol
