# Supplementary material for: Nonlinear connection between remnant cholesterol and stroke risk: evidence from the China health and retirement longitudinal study
Source: Lipids Health Dis. 2023 Oct 25;22:181. doi: 10.1186/s12944-023-01943-8 (PMC10601161; doi:10.1186/s12944-023-01943-8)
Supplement: Supplementary file 1 — Additional file 1: Table S1. The results of the collinearity screening. Table S2. The Baseline Characteristics of participants based on inflection point for RC. [file 12944_2023_1943_MOESM1_ESM.docx]

**Table S1** The results of the collinearity screening

|  | Step 1 |
| --- | --- |
| Gender | 1.9 |
| Age (years) | 1.3 |
| BMI (kg/m^2^) | 1.2 |
| Drinking status | 1.1 |
| Smoking status | 1.7 |
| Physical activity | 1 |
| Heart diseases | 1.1 |
| Hypertension | 1.2 |
| Diabetes mellitus | 1.1 |
| Lipid therapy | 1.1 |
| CRP (mg/L) | 1 |
| Scr (mg/dL) | 1.9 |
| Cystatin C (mg/L) | 1.9 |

**Table S2** The Baseline Characteristics of participants based on inflection point for RC

| Variables | RC group (mmol/L) | | P-value |
| --- | --- | --- | --- |
|  | <1.78 | ≥1.78 |  |
| Participants | 9562 | 505 |  |
| Gender |  |  | 0.128 |
| Male | 4478 (46.831%) | 219 (43.366%) |  |
| Female | 5084 (53.169%) | 286 (56.634%) |  |
| Age (years) | 59.232 ± 9.363 | 57.562 ± 8.935 | <0.001 |
| BMI (kg/m^2^) | 23.480 ± 3.944 | 25.664 ± 3.962 | <0.001 |
| Drinking status |  |  | 0.079 |
| Never drinkers | 1324 (13.846%) | 57 (11.287%) |  |
| Ever drinkers | 5851 (61.190%) | 303 (60.000%) |  |
| Current drinkers | 2387 (24.963%) | 145 (28.713%) |  |
| Smoking status |  |  | 0.050 |
| Never smokers | 5916 (61.870%) | 313 (61.980%) |  |
| Ever smokers | 806 (8.429%) | 57 (11.287%) |  |
| Current smokers | 2840 (29.701%) | 135 (26.733%) |  |
| Physical activity |  |  | 0.030 |
| No | 3446 (36.038%) | 206 (40.792%) |  |
| Yes | 6116 (63.962%) | 299 (59.208%) |  |
| Heart diseases |  |  | 0.010 |
| No | 8433 (88.193%) | 426 (84.356%) |  |
| Yes | 1129 (11.807%) | 79 (15.644%) |  |
| Lipid therapy |  |  | <0.001 |
| No | 9119 (95.367%) | 446 (88.317%) |  |
| Yes | 443 (4.633%) | 59 (11.683%) |  |
| Hypertension |  |  | <0.001 |
| No | 7278 (76.114%) | 328 (64.950%) |  |
| Yes | 2284 (23.886%) | 177 (35.050%) |  |
| Diabetes mellitus |  |  | <0.001 |
| No | 9058 (94.729%) | 453 (89.703%) |  |
| Yes | 504 (5.271%) | 52 (10.297%) |  |
| TG (mmol/L) | 1.335 ± 0.668 | 4.869 ± 2.022 | <0.001 |
| CRP (mg/L) | 1.000 (0.540-2.130) | 1.460 (0.830-2.870) | <0.001 |
| Scr (mg/dL) | 0.781 ± 0.239 | 0.806 ± 0.221 | 0.022 |
| FPG (mg/dL) | 108.602 ± 33.844 | 138.967 ± 54.750 | <0.001 |
| HbA1c (%) | 5.230 ± 0.780 | 5.562 ± 1.192 | <0.001 |
| Cystatin C (mg/L) | 1.009 ± 0.276 | 0.880 ± 0.307 | <0.001 |

Values are n (%) or mean ± SD or median (quartile)

RC: remnant cholesterol; BMI: body mass index; TG: triglycerides; Scr: serum creatinine; CRP: C-reactive protein; FPG: fasting plasma glucose; HbA1c: glycosylated hemoglobin
